# Nonpharmacological multicomponent intervention for mild cognitive impairment with a family-patient approach: protocol for the pilot clinical trial—INTERCOG study

**DOI:** 10.1186/s40814-026-01871-1

**Published:** 2026-06-27

**Authors:** Diana Carolina Tiga-Loza, William Armando Álvarez-Anaya, Raquel Rivera-Carvajal, Ariel Calderón Ardila, Jhosman Alfonso Buitrago-Buitrago, Alexander Pabon, Álvaro Castañeda-Hernández, Mabel Margoth Reyes-Pulido, Katherine Prado Guzmán, Hadder Uriel Acosta Salazar, Edwing Alberto Urrea-Vega, Lina María Carreño-Parra, Paul Anthony Camacho

**Affiliations:** 1 Instituto MASIRA, Facultad de Ciencias Médicas y de La Salud, Universidad de Santander, Bucaramanga, Colombia; 2 Doctorado en Enfermedades Infecciosas, Facultad de Ciencias Médicas y de La Salud, Universidad de Santander, Bucaramanga, Colombia; 3 Grupo de Investigación Salud, Rehabilitación y Trabajo SARET, Universidad Manuela Beltrán, Bucaramanga, Colombia; 4 Programa de enfermería, Universidad Cooperativa de Colombia, Bucaramanga, Colombia; 5 Grupo de Investigación Fisioterapia Integral, Facultad de Ciencias Médicas y de La Salud, Universidad de Santander, Bucaramanga, Colombia; 6 Programa de Fisioterapia, Grupo de investigación CliniUDES, Centro de Investigación Instituto MASIRA, Facultadde Ciencias Médicas y de la Salud, Universidad de Santander, Bucaramanga, Colombia; 7 Programa de Psicología, Grupo de investigación en calidad de vida y salud pública, Universidad Autónoma de Bucaramanga, Bucaramanga, Colombia; 8 Programa de Psicología, Grupo de Estudios Socio-Humanísticos, Facultad de Ciencias Sociales, Universidad de Santander, Bucaramanga, Colombia; 9 Direccion de Investigaciones, FOSCAL, Floridablanca, Colombia

**Keywords:** Cognitive dysfunction, Aged, Family member, Clinical trial, Multicomponent, Feasibility

## Abstract

**Background:**

Given the risk of progression to dementia ranging from 6 to 44.8% over approximately 4 years of follow-up, nonpharmacological multicomponent interventions are emerging as promising strategies to improve or maintain cognitive function and quality of life in individuals with mild cognitive impairment (MCI). This pilot study aims to evaluate the feasibility and potential efficacy of a multicomponent and transdisciplinary intervention focused on the dyad of older adults with MCI–family study partner to promote cognitive function in older adults.

**Methods:**

A pilot, randomized, controlled, and double-blind clinical trial will be conducted with 102 dyads consisting of an older adult with MCI and their family study partner. The participants will be randomly assigned to two groups: the intervention group (INTERCOG) and the control group. The INTERCOG group will receive a 12-week home-based intervention (twice weekly) with the support of a healthcare professional. The intervention components consist of cognitive training and stimulation, nutritional counseling, and physical exercises. Simultaneously, family members will participate in a training program on care and self-care. The control group will receive an Enhanced Standard of Care with educational messages on infection prevention. The feasibility of the study will be assessed through an analysis of recruitment, data collection, intervention acceptability, and implementation challenges; also, changes in global cognition and changes in specific cognitive domains will be measured as exploratory results. Secondary outcomes will include changes in health-related quality of life, caregiver burden, social support for family members, as well as changes in functional independence, frailty, physical capacity, family functioning, and nutritional status in patients, with follow-up at 3, 6, and 9 months. In a subsample of 40 participants, the diversity of the gut microbiota will be characterized, and the frequency of the APOE-ε4 allele will be determined.

**Discussion:**

Multicomponent and transdisciplinary interventions focused on the family member–patient dyad have the potential to optimize cognitive outcomes and quality of life. This pilot study will allow us to identify key aspects of the feasibility and potential efficacy of INTERCOG intervention, crucial information for the design of future larger-scale studies.

**Trial registration:**

NCT06408103. December 6, 2024, at clinicaltrials.gov. In recruitment.

**Supplementary Information:**

The online version contains supplementary material available at https://doi.org/10.1186/s40814-026-01871-1.

## Background

Population aging is expected to lead to an increase in chronic noncommunicable diseases, posing a challenge to health systems [1]. In the Americas, the global burden of dementia is projected to rise from 6.86 million cases in 2020 to 19.3 million in 2025, an escalation of more than threefold increase [2]. This projection underscores the need to implement comprehensive interventions that preserve cognitive function, prevent or slow the progression to dementia, and engage families, multiple sectors, and public policy [3].

Interventions focusing on individuals with mild cognitive impairment (MCI) are crucial, given that progression to dementia can occur in 6 to 44.8% of cases, with an average follow-up of 3.7 years in low- and middle-income countries [4]. Preventing this progression is vital in countries such as Colombia, where the prevalence of MCI in the elderly population is 17.93% [5]. This risk of progression is significantly compounded by existing comorbidities, nearly tripling in uncontrolled diabetes (HR = 2.87, 95% CI 1.20–6.85) and doubling with cardiac comorbidities (HR = 2.27, 95% CI 1.21–4.25) [6]. These figures underline the necessity of developing culturally relevant interventions that optimize cognitive function and mitigate progression to dementia by linking the prevention and control of noncommunicable chronic diseases. Furthermore, it is essential to integrate family study partners as active participants in the management process. Recent evidence indicates that caregiver burden is already present in family members of individuals with MCI and is significantly associated with the patient’s neuropsychiatric symptoms and the dyad’s quality of life, rather than being exclusively dependent on functional decline [7]. This integration is decisive because of the high caregiver burden often experienced by those caring for individuals with cognitive disorders, which negatively impacts their quality of life and can exacerbate the patient’s cognitive decline [8]. Multicomponent interventions have demonstrated efficacy in reducing the burden and increasing the social support of caregivers of patients with Alzheimer’s disease (AD) [9, 10].

Nonpharmacological multicomponent interventions (NPMIs) have been shown to be superior to single-domain interventions in improving cognition in patients with MCI. A meta-analysis of 28 studies published between 2011 and 2021 concluded that multidomain interventions had superior effects to single interventions in terms of global cognition (standardized mean difference SMD = 0.41, 95% CI, 0.23–0.59; *p* < 0.001) and other cognitive domains, such as executive functions, verbal fluency, and memory; however, high variability was observed in terms of the combination of interventions, duration, and frequency [11]. Similarly, another meta-analysis of 8 clinical trials indicated a moderate effect of NPMIs on global cognition, with a moderate effect for the Mini-Mental State Examination (MMSE) (SMD = 0.249, 95% CI, 0.067–0.431). Importantly, compared with cognitive-based interventions, studies that combine cognitive and physical interventions have shown superior effects on global cognition [12].

While recent years have seen the emergence of anti-amyloid immunotherapies as a potential pharmacological approach to modify MCI progression, their clinical implementation remains limited by high costs, rigorous eligibility requirements, and safety monitoring needs. Given the limited accessibility of these treatments in many global contexts and the significant risk of progression to dementia, the identification of modifiable risk factors is crucial for intervention.

Nutritional intake and diet are recognized lifestyle elements that are linked to the risk of MCI and AD [13]. Nutritional intervention has become a nonpharmacological approach, either through the intake of specific nutrients or dietary patterns that have protective effects [14, 15]. This protective effect is likely mediated by various biological pathways involved in cognitive impairment, such as amyloid-beta deposition, inflammation, and oxidative stress, which justifies the inclusion of nutritional counseling as a key component in a multicomponent intervention aimed at the management of MCI [15]. Additionally, nutritional interventions could modulate the composition of the gut microbiota, as various studies have shown differences between individuals with MCI/AD and healthy older adults [16, 17].

In line with the above, and despite the scientific support for NPMIs, the need to determine their optimal combination and dosage persists. This underscores the importance of comprehensive and multicomponent interventions that integrate physical activity, rehabilitation or cognitive stimulation, and nutritional counseling, also involving the family member as a future caregiver and a key factor for adherence. Therefore, this study aims to evaluate the potential efficacy and feasibility of a multicomponent and transdisciplinary intervention directed at the family study partner–older adult dyad with MCI to optimize cognitive function. For this purpose, the efficacy of the NPMIs on global and domain-specific cognitive function in patients with MCI will be explored. Additionally, the feasibility, acceptability, and adherence to the intervention will be examined. Additionally, changes in the health-related quality of life of the dyad, social support, independence, frailty, physical capacity, family cohesion and adaptability, nutritional status, and, in a subgroup, potential changes in the composition of the gut microbiota will be evaluated.

## Methods

### Design

We will conduct a randomized controlled pilot trial, parallel group, with an allocation ratio of 1:1, and investigator/evaluators will be blinded, following the SPIRIT guidelines and CONSORT extension for pilots [18, 19].

### Participants and eligibility criteria

Dyads, consisting of a patient diagnosed with MCI and a family study partner, who are both residents of four cities in the metropolitan area of Bucaramanga in Santander, Colombia, will be selected to participate in this study. Patients aged 55 years or older were required to have an MCI diagnosis made by a psychiatrist or neurologist and confirmed by neuropsychological evaluation, defined as a clinical stage that corresponds to the Mild Neurocognitive Disorder (mNCD) category in the DSM-5 [20] and ICD-11 (Code 6D81) [21]. Following the international consensus, the operational diagnosis was based on (1) objective evidence of modest decline in one or more cognitive domains and (2) preserved independence in daily life activities, although these may require greater effort or compensatory strategies [22]. In addition, they needed to have basic reading and writing skills, not present uncorrected sensory impairments (auditory or visual), have no limitations for physical activity or mobility, have a Barthel Index ≥ 80, a body mass index ≥ 18.5, tolerate oral intake, and be vaccinated against COVID-19 to mitigate the risk of potential infections during home-based intervention visits.

The exclusion criteria for patients in the study are as follows: a clinical history of congenital intellectual disability or psychosis; the presence of central nervous system pathologies that affect cognitive function; a diagnosis of untreated psychiatric disorders; clinically significant systemic diseases, including chronic kidney disease, hepatopathies, and lung diseases requiring oxygen therapy; active cancer treatment with chemotherapy; problematic use of alcohol, tobacco, or psychoactive substances; the need for nutritional support via oral, enteral, or parenteral routes; and the identification of low exercise tolerance, vertigo, or medical restriction for performing moderate to vigorous intensity physical activity. The study elimination criteria include institutionalization of the participant for a period longer than 1 week during the intervention phase, death of the participant during the intervention phase, and nonattendance at 34% or more of the scheduled sessions with professional accompaniment. The inclusion criteria for family study partner are as follows: (1) aged >18 years; (2) literate (basic reading/writing proficiency); and (3) a consanguineous or non-consanguineous kinship with the participant.

### Setting

The study will be conducted in Santander, Colombia, in four cities: Bucaramanga, Floridablanca, Girón, and Piedecuesta (metropolitan area). The participants will be recruited through three main sources: (1) healthcare providing institutions (IPSs) that collaborate with the study, including hospitals and health centers; (2) private neurology clinics; and (3) the general community through dissemination strategies such as press, radio, television, and personal contact. The Biomedical and Biotechnological Research Laboratory (LIBB) of the Bacteriology and Clinical Laboratory program at Universidad de Santander (UDES) will be used for sample collection for microbiota evaluation and APOE genotype analysis. These samples will be analyzed at the Universidad de Málaga. The design and implementation of the intervention will be carried out through a strategic alliance between the Universidad de Santander (UDES), Universidad Manuela Beltrán (UMB), Universidad Autónoma de Bucaramanga (UNAB), La Universidad Santo Tomás (USTA), Universidad de Málaga, and Fundación Oftalmológica de Santander (FOSCAL). The intervention will be carried out in the patients’ homes, and the family component will be provided through training at the UMB.

### Dyad involvement

**Fig. 1:**
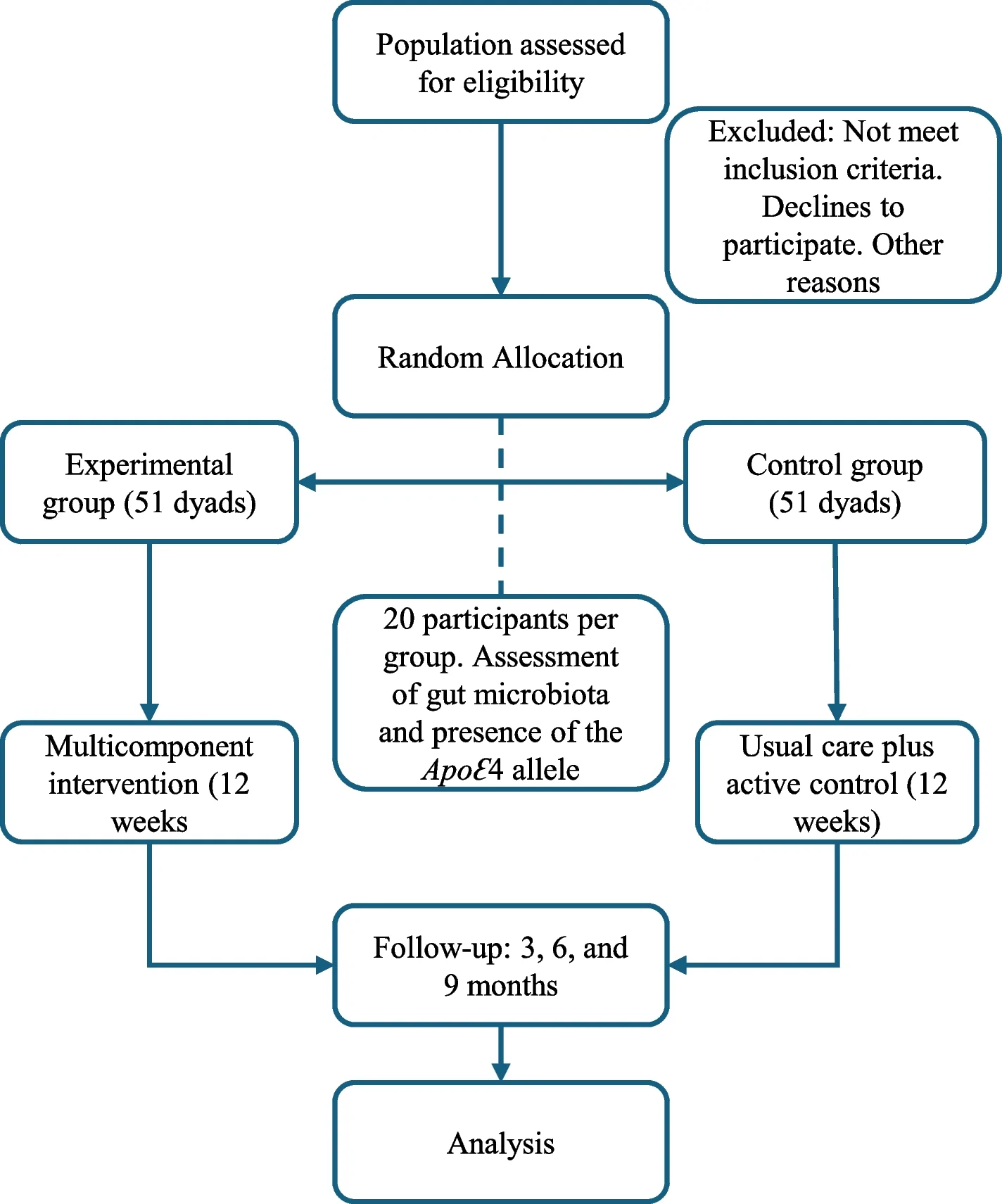
Flowchart of participant allocation

The call for participation in the study and the dissemination of inclusion criteria will be carried out in the three recruitment sources through informational posters, flyers, and announcements in radio and television media. Individuals who report amnestic cognitive complaints or have a diagnosis of MCI will be invited to participate, accompanied by a family study partner, and their interest will be expressed via WhatsApp messages or emails. A telephone interview will be conducted for the preselection of candidates, inquiring about their cognitive manifestations and the fulfillment of preliminary inclusion criteria, such as the ability to perform physical activities, the level of independence, cognitive complaints, and the presence of anxious or depressive symptoms. Following this, participants will be formally invited to participate in the study through an informed consent process for both the patient and their family member. Then, comprehensive baseline evaluations for the patient will be carried out by professionals in neuropsychology, nursing, physiotherapy, neurology, and psychiatry in appropriately equipped offices to ensure privacy, the absence of sound interference, and the availability of necessary resources. Similarly, family members will be asked to complete questionnaires regarding social support, quality of life, and caregiver burden. Finally, an adjudication committee composed of a neurologist, a psychiatrist, a neuropsychologist, and an epidemiological nurse will analyze the collected information to confirm the diagnosis of MCI according to the DSM-5 criteria and verify compliance with the established inclusion criteria (see Fig. 1).

### Intervention

INTERCOG is a nonpharmacological, multicomponent, and transdisciplinary intervention with a focus on the patient-family dyad. Its components include cognitive training and stimulation, physical training, nutritional counseling, and support for the family study partner. Patients assigned to the intervention group will receive the NPMI INTERCOG for 12 weeks, twice weekly home-based sessions and once weekly independent work session consisting of 10–30 min of daily walking and complete homework.

The home-based sessions will include cognitive training/stimulation, physical training, and nutritional counseling. For their part, family study partners will receive weekly training at a university institution for 12 weeks. The intervention structure for the dyad is described in Table 1. To facilitate the intervention process, two workbooks were developed (one for patient and another for family member) [23, 24], as were video recordings to guide physical activity and nutritional recommendations [25]; detailed 12-week schedule of the intervention is provided in Supplementary Table S1. A distinctive feature of the INTERCOG program is its home-based delivery model. By conducting the intervention within the participants’ domestic environment, we aim to reduce geographical and economic barriers to care—such as travel time and transportation costs—which are often deterrents for older adults and their family study partners in regions like Santander.

**Table 1:** The nonpharmacological, multicomponent intervention INTERCOG for MCI focuses on the patient–family dyad, Bucaramanga, and the metropolitan area, Santander, Colombia, 2025

Component	Target domains/clinical goals	Enhanced specific activity examples	Tailoring and progression
Cognitive	Executive function, memory (episodic/working/sensory), attention, calculation, and orientation	Ecological task planning: organizing medication schedules and grocery lists. Neurocognitive training: life-history reconstruction (autopsychic orientation), trail making (visual attention), and arithmetic logic puzzles for mental calculation	3 levels of difficulty per activity (scaffolded progression). Tasks advance from simple stimulus identification to complex multi-step problem solving
Physical	Aerobic capacity, muscular strength, static/dynamic balance, and joint mobility and simultaneous dual task	Dual-task integration: performing verbal fluency tasks while walking. Proprioceptive challenge: lower-limb strength exercises (e.g., squats) performed with eyes open/closed to increase neuro-vestibular demand. Standardized 21-video series for strength, gait, and balance	Progression by intensity: workload is monitored via the Modified Borg Scale, increasing from a baseline of intensity 4 (month 1) up to intensity 8 (month 3)
Nutritional	Glycemic control, brain-health nutrients (omega-3/antioxidants), and dietary habit modification	Interactive nutritional workshops: “cooking laboratories” for preparing functional foods (e.g., quinoa, flaxseed). Behavioral coaching: strategies for sugar reduction and identifying ultra-processed vs. whole-grain carbohydrates	Shift from theory to practice: transitions from basic macronutrient education to the implementation of Mediterranean-style neuroprotective diets in daily life
Familiar	Psychoeducation, communication skills, and prevention of caregiver burden	Empowerment sessions: family reorganization workshops, conflict resolution training, and relaxation therapies. Technological literacy: training on the use of health-monitoring apps and virtual support networks	Focus on resilience: moves from initial program induction and emotional support to the establishment of long-term sustainable caregiving routines

The control group, in addition to usual care, will receive weekly information on the prevention of infectious diseases on their mobile devices. This material, similar to that available online, will be provided as an Enhanced Standard of Care (ESOC) (Supplementary Table S2). Once the study is completed, participants in the control group will receive an intensive 1-week version of the NPMI INTERCOG, as well as the workbooks for the patient and the family member.

### Intervention fidelity and standardization

The INTERCOG intervention will be delivered by a trained multidisciplinary team (nurses, psychologists, physiotherapists, and speech-language pathologists). To minimize inter-interventionist variability, the program utilizes two registered workbooks [23, 24], which contain all standardized cognitive, nutritional counseling, physical, and familiar activities. Prior to the trial, all interventionists will undergo a standardized training program focused on the theoretical foundations of the INTERCOG model and the practical application of the protocols. This training is supported by an instructional video series consisting of 21 standardized physical training sessions (one per session) and 9 videos about nutritional advice. To facilitate real-time access during home visits, the workbooks include integrated QR codes that link directly to the corresponding video demonstrations, ensuring uniform delivery of the exercise components [25].

Fidelity is maintained through a multi-layered strategy: (1) digital process logs: interventionists will use REDCap to record session attendance and qualitative execution reports in real-time; (2) clinical supervision: weekly team meetings to discuss implementation challenges; and (3) direct oversight: co-investigators will perform unannounced home visits and follow-up calls to ensure protocol adherence.

### Outcomes

To evaluate the feasibility, acceptability, and adherence of the intervention as a primary outcome, several dimensions proposed by Gadke et al. [26] will be assessed. Table 2 presents the dimensions, questions, measurements, and possible actions to be taken in the progress of the trial.

**Table 2:** Evaluation of feasibility, acceptability, and adherence of NPMI INTERCOG

Dimension	Questions	Measurements	Possible actions
Recruitment and retention capability	What are the difficulties or ease in recruiting dyads? Is it possible to recruit 102 dyads that meet the inclusion criteria?	Number of collaborating institutions contacted, the number of interested individuals, the number of individuals preselected and evaluated, the participant rejection criteria, the number of people who decided not to continue in the trial, and the recruitment rate	Expanding the number of collaborating institutions and ways to disseminate the call for participation or extend it overtime, increasing recruitment staff, and strategies to prevent participants dropouts
Data collection procedures	Are the measurements sensitive enough to determine change? Are the measurement procedures useful? Were any outcomes missing? What are the difficulties or ease in data collection?	Difficulties in data collection, changes in questionnaire cutoff points, completeness of data collection, data collected unnecessarily	Supervision of instrument completion and data quality control Alternative analysis of the variable without cutoff points, or as a continuous variable, or establishment of tertiles or quartiles
Social validity	Do participants perceive the intervention as appropriate, safe, and potentially effective? Are the frequency, intensity, and methods used in the interventions perceived as appropriate? What are the reasons for not participating in an intervention?	Participant surveys and interviews to obtain postintervention feedback data in person and by telephone	Implement strategies to improve understanding of interventions Evaluate the possibility of making any adjustments to the intervention methodology
Practicality	Can interventions be implemented with time, training, available resources/materials?	Interview participants and healthcare professionals to identify implementation challenges related to practicality Availability to acquire materials and supplies for the intervention	Evaluate alternative materials or resources that are easier to acquire, reduce or recalculate intervention times, and retrain staff
Adherence	Do participants comply with the proposed activities?	Completion rate Activity completeness	In the event of difficulties, interview the possible reasons to evaluate alternatives for compliance

Also, the potential efficacy of the NPMI INTERCOG will be evaluated as an exploratory outcome by measuring the change in global and cognitive domains: (1) global cognition: change in the scores of the Montreal Cognitive Assessment (MoCA) as a primary cognitive endpoint [27, 28] and the Mini-Mental State Examination (MMSE) [29, 30]; (2) verbal learning and memory: change in the scores of the Hopkins Verbal Learning Test (HVLT) [31, 32]; (3) processing speed and visual attention: change in scores on the Symbol Digit Modalities Test (SDMT) [32, 33]; (4) visual attention, processing speed, and visuospatial ability: change in the scores of the Trail Making Test (TMT-Forms A and B) [32, 34]; (5) executive function and selective attention: change in the scores of the Stroop Color and Word Test [35, 36]; (6) perceptual organization and visual memory: change in the scores of the Rey–Osterrieth Complex Figure Test [32, 37]; (7) verbal fluency and executive functions: change in the scores of the Semantic and Phonemic Verbal Fluency Test [32]; (8) comprehension and expression levels: change in the scores of the Boston Naming Test [32, 38]; (9) verbal comprehension: change in the scores of the Token Test [39, 40]; (10) digit span: change in the scores of the digit span subtest of the WAIS-III (Wechsler Adult Intelligence Scale-Third Edition) [41]. Descriptions of the measures, scores, cutoff points, and interpretations are described in the trial registry [42].

Among the secondary outcomes, the variation in health-related quality of life will be estimated through the application of the 36-Item Short Form Health Survey (SF-36) to both the patient and the family member [43, 44]. As a primary functional endpoint, we stablished the change in instrumental activities of daily living (IADL) measured through the Lawton and Brody scale [45, 46]; also, the change in the Fried’s criteria of frailty, which includes the assessment of weight loss (weight, body mass index, and waist-to-hip ratio), physical activity, exhaustion, and grip strength, will be evaluated [47, 48]. Nutritional status with the Mini Nutritional Assessment (Long-MNA) [51], and the change in the risk of falls will be evaluated with the Tinetti balance scale [52].

### Data collection procedures

Data collection will be conducted at three time points: (1) during the preselection process, (2) at baseline, and (3) during follow-up (at 3, 6, and 9 months of the study). At each point, a team of professionals from various previously trained disciplines will administer multiple questionnaires and perform measurements. The detailed schedules of the instruments, measurement time points, and evaluators are presented in Table 1.

### Preselection

To identify compliance with the inclusion criteria, the following instruments will be used in the preselection stage: Geriatric Depression Scale (GDS): patients with a score below 10 points will be invited to participate [53]. Barthel Index of Activities of Daily Living (BI-ADL): independence in performing activities of daily living will be evaluated, establishing a cutoff point of 80 or more points to confirm functional independence compatible with the diagnosis of MCI [54]. Physical Activity Readiness Questionnaire for Everyone (PAR-Q & YOU): this will be used to identify the absence of restrictions for physical activity, considering negative responses to 6 of the 7 questions as a participation criterion (the question about medication use will be excluded, considering it a precaution and not a restriction) [55].

### Baseline and postintervention follow-up (0, 3, 6, and 9 months)

Neuropsychological assessment: a neuropsychologist will administer tests to evaluate global cognition, and the cognitive domains will be defined as the primary outcome. Together, these tests provide a detailed profile of the key cognitive areas affected by MCI.

Nursing assessment: measurements of blood pressure and pulse will be taken with an OMROM model HEM-7130 automated monitor. In addition, the following instruments will be administered: the SF-36 Health Survey [49], Patient Characterization Form (basic information, family, pathological, pharmacological, and surgical history), Family APGAR [50], Mini Nutritional Assessment (Long-MNA®) [51], Pittsburgh Sleep Quality Index (PSQI) [52], and Bristol Stool Form Scale (BSFS) [53].

Physiotherapy assessment: dominant handgrip strength will be measured with a JAMAR 6386 hydraulic analog hand dynamometer. Weight and body composition will be assessed by bioimpedance with a Body Composition Monitor and Scale OMRON HBF 514C. Height and waist and hip circumferences will be recorded via a standardized method. Additionally, Tinetti gait and balance assessment tool [54], Senior Fitness Test [55], Linda Fried Frailty Phenotype [47], and Lawton-Brody Instrumental Activities of Daily Living Scale [46] will be administered.

Family member assessment: nursing staff will apply the GCPC-UN-D© sociodemographic form for the characterization of study partner [56]. Social support will also be assessed via the Medical Outcomes Study Social Support Survey (MOS) [57], quality of life with the SF-36 [49], and caregiver burden with the Zarit Caregiver Burden Interview [58, 59] (see Supplementary Table S3 for more details).

### Data management and monitoring

Study data will initially be collected via paper forms and subsequently double-entered into REDCap electronic data capture tools, hosted at the Universidad de Santander [60]. To ensure data integrity, a researcher will audit the database for completeness and resolve discrepancies identified during the double-entry process.

Healthcare professionals delivering the intervention will be trained to recognize and report any potential adverse events (AEs), particularly those related to the physical activity component. All AEs will be immediately reported to the principal investigator and the study’s insurance provider, as detailed in the protection policy.

### Sample size

The required sample size was estimated to be 102 dyads, comprising 51 dyads per group (1:1 ratio), based on a repeated-measures design and two correlated means. The calculation was performed using an alpha level of 5% and a statistical power of 80%, adjusting for a 15% estimated attrition rate. The effect size data (MoCA: 0.7; MMSE 0.5) and the standard deviation of differences (MoCA: 1.8; MMSE 2.3) were derived from previous studies [61, 62]. All sample size calculations were executed using STATA-13 statistical software.

### Exploratory analysis: gut–brain axis and APOE genotype

To evaluate the biological plausibility of the INTERCOG intervention, an exploratory analysis of the gut–brain axis will be conducted on a targeted subsample of the first 40 participants (20 per group) diagnosed with amnestic mild cognitive impairment (aMCI). This phenotypic subset is analyzed not as a separate study, but as a mechanistic component intended to identify potential biological mediators that may underpin the clinical changes in cognitive function. Given that aMCI carries the highest risk of progression to Alzheimer’s disease and that the APOE-ε4 allele acts as a critical genetic moderator of neurodegeneration, characterizing these markers is essential to understanding the differential response to the multicomponent treatment.

To ensure the validity of the microbiota analysis and minimize confounding factors, specific exclusion criteria apply strictly to this subsample: (1) continuous use of probiotics, prebiotics, antibiotics, antifungals, or antivirals within the 3 months prior to enrollment; (2) a clinical diagnosis of inflammatory bowel diseases (such as Crohn’s disease or ulcerative colitis) or a history of surgical resection of the intestinal tract; and (3) the use of corticosteroids or immunosuppressants in the 30 days preceding the study. These microbiome-specific restrictions do not limit eligibility for the primary clinical trial, thereby preserving the representativeness of the main study sample.

For the statistical analysis of the microbiota data, alpha diversity differences will be evaluated via the Wilcoxon random-sum test, beta diversity variations will be assessed via permutational multivariate analysis of variance, and bacterial differences between the comparison groups will be identified via linear discriminant analysis effect size.

### Randomization and allocation concealment

A two-level stratified block randomization scheme will be employed to ensure balance across key demographic variables, given the characteristics of the screened population. The randomization sequence will be generated by a researcher independent of the core study team using the blockrand module (version 1.5) in R software (version 4.4.3). This individual will not participate in any study procedures.

The stratification process will include the following: first-level (stratification) dyads will be stratified into four groups based on two factors: sex (male/female) and age (above/below 71 years). Second level (block size): within each stratum, a block size of 2 is used to ensure an automatic 1:1 allocation ratio between the intervention and control groups.

The allocation sequence will be concealed, and the assignment will be executed in two phases: first, after the initial 52 dyads are completely identified and screened, and second, again for the remaining 50 dyads. This approach ensures that all participants maintain the same probability of assignment and helps maintain balance throughout the recruitment process. This entire procedure will be performed by personnel external to the core research team.

### Blinding

Owing to the nature of the intervention, which cannot be masked with a placebo, it is not possible to blind the participants, their family members, or the intervention team. However, to minimize detection and performance bias, blinding will be strictly maintained at the assessment and analytical levels. The assessment team and data collectors will remain operationally independent from the intervention staff throughout the study. Furthermore, participants will be requested at each follow-up visit to refrain from discussing their assigned activities with the evaluators. Finally, statistical analysis will be performed by an analyst blinded to group allocation; data will be managed using arbitrary codes, which will only be unmasked upon completion of the primary analysis.

### Statistical methods

Continuous data will be described using means and standard deviations (SDs) or medians and interquartile ranges (IQRs), depending on their distribution as assessed by the Shapiro–Wilk test. These data will be compared between the intervention and control groups via Student’s *t*-test or the Mann–Whitney *U* test, respectively. Categorical data will be summarized using absolute and relative frequencies and compared between groups via the *χ*^2^ test or Fisher’s exact test, as appropriate.

Outcomes will be analyzed across the baseline, 3-, 6-, and 9-month follow-up measurements via generalized linear mixed-effects models (GLMMs). These models will include fixed factors (intervention group, time, and the group × time interaction) and a random effect for participants to account for individual variability. The models utilize maximum likelihood estimation (MLE) methods to handle data that are missing at random (MAR). Potential confounding variables identified during the descriptive analysis were adjusted for. To account for the potential confounding effect of social interaction, perceived social support (measured via the MOS survey) will be included as a time-varying covariate in the models. The results are reported as adjusted mean differences with their respective 95% confidence intervals (95% CIs). Imputation methods will not be used.

Analysis will be performed on both an intention-to-treat (ITT) and per-protocol (PP) basis. To account for multiplicity and reduce the risk of type I error across the various secondary outcomes, *p *values will be adjusted using the Benjamini–Hochberg false discovery rate (FDR) procedure. The level of statistical significance will be set at *α* = 0.05. All statistical analyses will be conducted via STATA 17 (Stata Corporation, College Station, USA).

### Feasibility analysis and success thresholds

The NPMI INTERCOG viability will be determined by three quantitative benchmarks: recruitment (>70% of eligible candidates), retention (>70% at 9 months), and adherence (>70% session completion). These thresholds account for the high participant burden inherent in multicomponent lifestyle interventions especially in geriatric populations, where age-related attrition is significant factor [63, 64]. Additionally, qualitative acceptability will be evaluated through thematic analysis of semi-structured interviews; also, we will obtain an average satisfaction score (0–5 points) on the participant feedback surveys. The intervention will be deemed feasible for a definitive trial if at least two quantitative targets are met alongside positive qualitative feedback.

### Ethics and dissemination

The overarching INTERCOG study (which includes the review phase, intervention design, and clinical trial pilot study) was initially approved by the Institutional Bioethics Committee of Universidad de Santander (Acta No. 013, May 24, 2022). Specific ethical approval for the pilot clinical trial phase has been granted by the CEI—the FOSCAL Research Ethics Committee (Approval No. 11319/2025, June 25, 2025), the Hospital Universitario Los Comuneros Research Ethics Committee (February 20, 2025), and the Instituto de Salud de Bucaramanga ISABU (Registration No. 00004601, December 10, 2024). Permissions have also been secured from all the collaborating medical centers and hospitals involved in recruitment. Any necessary changes to the protocol will be promptly reported to the Research Committees of UDES and FOSCAL.

The participants who were interested in the microbiota and ApoE analysis provided additional written informed consent. This extra consent was required because this specific component of the research was approved after, following the acquisition of a second funding source, and necessitated independent ethical review. Ethical approval was granted by the Institutional Bioethics Committee of Universidad de Santander (Acta No. 03 del 8 de abril del 2024).

Written informed consent will be obtained from both the patient and their family member. The consent process will involve a detailed explanation of the research objectives, the reason for the invitation to participate, the procedures, potential risks, and benefits (if any), and participants will be given ample opportunity to clarify any questions they may have. The information supplied by or obtained from participants will be treated as confidential. Data will be stored in a manner that preserves individuals’ rights to privacy and intimacy. Access to the data, housed in REDCap, will be restricted to using authorized usernames and passwords managed by the study coordination, and access time will be limited. Participants assigned to the control group will receive the full NPMI intensively for 1 month (twice weekly) at the conclusion of the study (after the 9-month follow-up), as compensation for their participation. No participant will receive monetary compensation.

## Discussion

The management of cognitive decline represents a substantial challenge, particularly in Latin American countries with progressive and accelerated population aging [65]. NPMIs are promising targets for maintaining or improving global cognitive function [11, 12], physical status, and quality of life in older adults with MCI and potentially preventing progression to dementia [66]. Given that better outcomes are associated with greater adherence [67], the primary focus of this study is the evaluation of the feasibility and implementation of the NPMI INTERCOG. Despite increasing evidence, challenges persist regarding the generalization and implementation of these interventions, due to the lack of standardization in their duration, intensity, and frequency [66]. A key strength of the NPMI INTERCOG is its comprehensive scope, which addresses not only global cognition or specific cognitive domains but also critical health dimensions such as functional independence, physical frailty, quality of life, and nutritional status.

The home-based approach of this trial aligns with the growing need for scalable and inclusive healthcare solutions. Unlike traditional clinic-based programs, home-delivered care enhances ecological validity by fostering behavioral changes in the exact setting where they must be sustained. This model not only facilitates recruitment and retention but also empowers the dyad, transforming the home into a therapeutic space that supports cognitive health without the stress associated with clinical environments.

Furthermore, the inclusion of family members is fundamental for proactively mitigating future caregiver burden and increasing perceived social support. We anticipate that linking the family will also enhance adherence to multicomponent interventions. The execution of this pilot will provide critical information for future implementations or larger-scale clinical trials by estimating recruitment and adherence rates, dropout factors, and specific implementation difficulties encountered by the intervention team. Additionally, this study offers a unique opportunity to explore the links between the gut–brain axis and variables related to the gut microbiota and evaluate the presence of ApoE risk genotypes.

Although recruitment from specific geographical areas may restrict generalizability and the intervention duration and follow-up period were designed for a pilot, the results will be important for large-scale clinical trials.

This pilot trial has certain limitations. First, while the study aims to evaluate feasibility, the sample size is only powered to assess potential cognitive efficacy; thus, results should be considered exploratory and regarding microbiota, and APOE outcomes may not be generalizable to all MCI subtypes. Additionally, while conducting the study in Santander, Colombia, strengthens its ecological validity within this specific cultural and nutritional setting, the intervention may require adaptation before being translated to different geographic or socioeconomic contexts.

## Supplementary Information


Supplementary Material 1.

## Data Availability

No datasets were generated or analysed during the current study.

## Abbreviations

AD: Alzheimer’s disease
AEs: Adverse events
aMCI: Amnestic mild cognitive impairment
APGAR: Adaptability, partnership, growth, affect, and resolve—family functioning
APOE: Apolipoprotein E
BI-ADL: Barthel Index of Activities of Daily Living
GCPC-UN-D: Guide to Characterizing the Chronic Patient and their Caregiver—Universidad Nacional de Colombia
GDS: Geriatric Depression Scale
GLMMs: Generalized linear mixed-effects models
HR: Hazard ratio
HVLT: Hopkins Verbal Learning Test
IADL: Instrumental activities of daily living
IQR: Interquartile range
ITT: Intention-to-treat
MAR: Missing at random
MCI: Mild cognitive impairment
METs: Metabolic equivalents
MLE: Likelihood estimation
MMSE: Mini-Mental State Examination
MNA: Mini Nutritional Assessment
MoCA: Montreal Cognitive Assessment
MOS: Medical Outcomes Study Social Support Survey
INTERCOG: Intervención no farmacológica multicomponente para la cognición del adulto mayor
NPMI: Nonpharmacological multicomponent intervention
PP: Per-protocol
SD: Standard deviation
SDMT: Symbol Digit Modalities Test
SF-36: 36-Item Short Form Health Survey
SMD: Standardized mean difference
TMT: Trail Making Test
WAIS-III: Wechsler Adult Intelligence Scale-Third Edition
95% CI: 95% Confidence interval
