# Supplementary material for: Nonpharmacological multicomponent intervention for mild cognitive impairment with a family-patient approach: protocol for the pilot clinical trial—INTERCOG study
Source: Pilot Feasibility Stud. 2026 Jun 27;12:127. doi: 10.1186/s40814-026-01871-1 (PMC13523507; doi:10.1186/s40814-026-01871-1)
Supplement: Supplementary file 1 [file 40814_2026_1871_MOESM1_ESM.docx]

Supplementary Table S1

A comprehensive 12-week Schedule of NPMI INTERCOG

| **Month / Week** | **Type of delivery** | **Component** | **Theme / Domain** | **Specific Activity** | **Volume / Intensity / Duration** |
| --- | --- | --- | --- | --- | --- |
| Month 1 / Week 1 | Home session 1 | Cognitive | Autopsychic Orientation | Life History Reconstruction: Narrative recall of personal milestones. | 20 min |
|  |  | Nutritional | Macronutrients | Sugar Identification: Strategies to reduce added sugars in industrial beverages. | 15 min |
|  |  | Physical | Aerobic / Mobility | Warm-up, Passive Gymnastics, and Stretching. | 2 sets / 8 reps (Borg: 4). Total: 15 min |
|  | Home session 2 | Cognitive | Autopsychic Orientation | Genogram Construction: Visual mapping of family structure and roles. | 15 min |
|  |  | Nutritional | Metabolism | Sugar Withdrawal Management: Physiological education on sugar reduction. | 15 min |
|  |  | Physical | Balance / Equilibrium | Joint mobility and controlled breathing exercises. | 2 sets / 8 reps (Borg: 4). Total: 15 min |
|  | Independent Tasks | All | Self-Managed | Evidence of Genogram completion; 10-minute daily walk; continued sugar reduction. | Freq: 5x/week (Borg: 4) |
|  | Group session | Familiar | Induction / Communication | The Art of Expression: Family reorganization and emotional induction to the program. | 180 min |
| Month 1 / Week 2 | Home session 1 | Nutritional | Habit Modification | Identifying Ultra-processed Foods: Reducing "junk food" and snack consumption. | 15 min |
|  |  | Cognitive | Attention & Concentration | Selective Attention Training: Focus and cancellation tasks. | 15 min |
|  |  | Physical | Muscular Strength | Targeted strength training and breathing control. | 2 sets / 8 reps (Borg: 4). Total: 15 min |
|  | Home session 2 | Nutritional | Healthy Alternatives | Functional Snacking: Options for nutrient-dense between-meal snacks. | 15 min |
|  |  | Cognitive | Dual-Tasking | Cognitive-Motor Stimulation: Simultaneous attention tasks during physical movement. | 30 min (combined) |
|  |  | Physical | Aerobic / Balance | Color-coded target stepping and coordination drills. | 2 sets / 9 reps (Borg: 4). Total: 20 min |
|  | Independent Tasks | Independent Tasks | Self-Managed | Vowel Cancellation Task: Selecting "a" in a text (attention); 15-minute daily walk. | Freq: 5x/week (Borg: 4) |
|  | Group session | Familiar | Support Networks | Helping Hands: Identification of social support networks and baseline template construction. | 165 min |
| Month 1 / Week 3 | Home Session 1 | Nutritional | Dietary Diversification | Grain Substitution: Reducing wheat-based products and incorporating ancient grains (e.g., Quinoa, Amaranth). | 15 min |
|  |  | Cognitive | Memory I | Working & Episodic Memory Training: Recall tasks and structured cognitive stimulation. | 15 min |
|  |  | Physical | Muscular Strength | Resistance Training: Warm-up, targeted strength exercises (2 sets / 8 reps; 30s rest), and stretching. | Borg: 4. Total: 20 min |
|  | Home Session 2 | Interdisciplinary | Multicomponent Integration | Culinary workshop (Quinoa): Hands-on preparation of functional foods integrated with simultaneous attention and coordination tasks. | Borg: 4. Total: 65 min |
|  | Independent Tasks | All | Self-Managed / Memory | Auditory Narration Task: Recording a voice note describing the cooking experience; submitting photo evidence of healthy meals via WhatsApp. | Walk: 10 min (Borg 4), 5x/week |
|  | Group Session | Familiar | Resilience & Habits | Support Networks: Promoting healthy lifestyle habits and constructing a formalized caregiver support framework. | 165 min |
| Month 1 / Week 4 | Home Session 1 | Nutritional | Complex Carbohydrates | Whole Grain Identification: Differentiating between simple and complex carbohydrates in the local diet. | 15 min |
|  |  | Cognitive | Attention & Concentration II | Selective and Sustained Attention Training: Advanced neurocognitive drills. | 20 min |
|  |  | Physical | Balance / Coordination | Aerobic & Gait Stability: Target stepping (4 sets / 8 reps; 30s rest) with directional changes. | Borg: 4. Total: 30 min |
|  | Home Session 2 | Nutritional | Cultural Adaptation | Traditional Colombian Plate: Re-engineering local traditional dishes into healthy, balanced versions. | 15 min |
|  |  | Cognitive | Executive Function | Executive Control Training: Simultaneous cognitive-motor tasks focused on planning. | 30 min (combined) |
|  |  | Physical | Core Strength | Abdominal & Postural Stability: Targeted strength exercises (4 sets / 8 reps; 30s rest). | Borg: 4. Total: 30 min |
|  | Independent Tasks | Cognitive and nutritional | Executive Function | Trail Making Task: Completion of paper-and-pencil connectivity drills; photo evidence of traditional meal preparation. | Walk: 20 min (Borg 4), 5x/week |
|  | Group Session | Familiar | Digital Literacy | Technology for Care: Training on health-monitoring apps, digital support tools, and healthy habits. | 180 min |
| Month 2 / Week 5 | Home Session 1 | Nutritional | Lipids & Brain Health | Healthy Fats Identification: Functions and sources of essential fatty acids for neuroprotection. | 15 min |
|  |  | Cognitive | Mental Calculation | Arithmetic Logic Training: Interactive numerical puzzles and mathematical operations. | 20 min |
|  |  | Physical | Balance / Proprioception | Proprioceptive Challenge: Squats performed with eyes open/closed (4 sets / 6 reps). | Borg: 6. Total: 35 min |
|  | Home Session 2 | Physical and cognitive | Cognitive-Motor Integration | Combined Strength & Logic: Resistance training (4 sets / 8 reps) integrated with simultaneous calculation tasks. | Borg: 6. Total: 70 min |
|  | Independent Tasks | All | Self-Managed Practice | Mathematical Operations: Summation and logic sheets; healthy fat substitution evidence (WhatsApp). | Walk: 20 min (Borg 6), 5x/week |
|  | Group Session | Familair | Resilience & Coping | Conflict Resolution Workshop: Human communication skills and relaxation therapy sessions. | 180 min |
| Month 2 / Week 6 | Home Session 1 | Nutritional | Culinary Techniques | Healthy Cooking Methods: Reducing trans fats through improved meal preparation techniques. | 10 min |
|  |  | Cognitive | Sensory & Working Memory | Sensory Memory Stimulation: Recognition and recall of sensory stimuli (visual, auditory, tactile). | 20 min |
|  |  | Physical | Postural Stability | Static and Dynamic Stability: Multi-directional balance drills (8 sets total). | Borg: 6. Total: 35 min |
|  | Home Session 2 | Cognitive | Memory II | Sensory Memory Training: Integration of sensory recall within daily living tasks. | 20 min |
|  |  | Physical | Muscular Strength | Progressive Resistance: Targeted upper and lower body strength (2 sets / 10 reps). | Borg: 6. Total: 35 min |
|  | Independent Tasks | All | Sensory Integration | Sensory Description Task: Written record of environmental stimuli perceived during walks; recipe evidence. | Walk: 20 min (Borg 6), 5x/week |
|  | Group Session | Familiar | Cognitive Stimulation | The Art of Remembering: Training caregivers in memory-enhancing strategies for home routines. | 160 min |
| Month 2 / Week 7 | Home Session 1 | Nutritional | Omega-3 Fatty Acids | Omega-3 Supplementation: Practical ways to include flaxseed, fish, and nuts in the diet. | 15 min |
|  |  | Cognitive | Language & Fluency | Verbal Fluency Training: Lexical and semantic retrieval tasks. | 20 min |
|  |  | Physical | Muscular Strength | Advanced Resistance Training: 4 sets / 8 reps of functional movement patterns. | Borg: 6. Total: 40 min |
|  | Home Session 2 | All | Functional Nutrition | Culinary Laboratory (Flaxseed Beverage): Integrated preparation of neuroprotective drinks with cognitive-motor drills. | Borg: 6. Total: 90 min |
|  | Independent Tasks | All | Narrative Memory | Saying/Proverb Completion Task: Idiom-based cognitive sheets; submission of flaxseed recipe photos. | Walk: 20 min (Borg 6), 5x/week |
|  | Group Session | Familiar | Social Participation | Social Bonding: Dance therapy and family relationship-building workshops. | 160 min |
| Month 2 / Week 8 | Home Session 1 | Nutritional | Healthy Fat Sources | Classification of Lipids: Identifying healthy vs. inflammatory fats in local products. | 15 min |
|  |  | Cognitive | Visuoconstruction | Visuospatial Training: Pattern recognition and structural assembly tasks. | 15 min |
|  |  | Physical | Dynamic Balance | Advanced Gait Drills: Progressive distance gait tasks (3 series). | Borg: 6. Total: 40 min |
|  | Home Session 2 | Cognitive | Visuoconstruction | Visuoconstructive Training: Advanced spatial reasoning and graphic construction. | 20 min |
|  |  | Physical | Aerobic Capacity | Static High-Intensity Gait: High-tempo stepping (3 sets / 7 min). | Borg: 6. Total: 40 min |
|  | Independent Tasks | All | Executive Planning | Routine Management: Scheduling avocado/nut intake; establishing a structured daily routine sheet. | Walk: 20 min (Borg 6), 5x/week |
|  | Group Session | Familiar | Sustainable Care | Community Support: Strengthening family and social networks for long-term care sustainability. | 160 min |
| Month 3 / Week 9 | Home Session 1 | Nutritional | Antioxidants | Neuroprotection: Benefits of fruits, vegetables, and legumes in reducing oxidative stress. | 15 min |
|  |  | Cognitive | Praxias / Gnosias | Visuospatial Recognition: Complex gnosis and motor planning (praxis) exercises. | 15 min |
|  |  | Physical | Aerobic / Rhythm | Dance Therapy: Rhythmic flexo-extension movements (3 sets / 6 min). | Borg: 8. Total: 40 min |
|  | Home Session 2 | Nutritional | Food Association | Dietary Patterns: Associating specific antioxidants with daily meal preparation. | 15 min |
|  |  | Cognitive | Executive Function | Intermediate Puzzle Solving: Advanced visuospatial reasoning and planning. | 20 min |
|  |  | Physical | Core Strength | Advanced Resistance: High-intensity abdominal and strength work (2 sets / 10 reps). | Borg: 8. Total: 45 min |
|  | Independent Tasks | All | Geometric Logic | Geometric Pattern Completion: Cognitive sheets; 30-minute walk; consumption of 2 fruits/day. | Walk: 30 min (Borg 8), 5x/week |
|  | Group Session | Familiar | Leisure & Relaxation | Energy Recharge: Identification and implementation of outdoor leisure activities and relaxation techniques. | 160 min |
| Month 3 / Week 10 | Home Session 1 | Nutritional | Leafy Greens | Green Vegetable Benefits: Role of folate and vitamin K in cognitive maintenance. | 15 min |
|  |  | Cognitive | Alopsychic Orientation | Spatial & Temporal Awareness: Advanced orientation drills related to the external environment. | 20 min |
|  |  | Physical | Balance / Gait | Dynamic Trunk Displacement: High-intensity gait with postural challenges (4 sets / 3 min). | Borg: 8. Total: 50 min |
|  | Home Session 2 | Nutritional | Cognitive Association | Nutrient-Cognition Link: Identifying vegetables that support executive functions. | 15 min |
|  |  | Cognitive | Alopsychic Orientation | Environmental Navigation: Applying orientation skills to real-world scenarios. | 20 min |
|  |  | Physical | Strength / Balance | Static Gait & Squats: High-intensity lower-limb strength (3 sets / 10 reps). | Borg: 8. Total: 45 min |
|  | Independent Tasks | All | Autobiographical Memory | Past & Present Mapping: Writing about 4 visited locations (2 past, 2 present); 30-minute free-choice walk. | Walk: 30 min (Borg 8), 5x/week |
|  | Group Session | Familiar | Healthy Routines | Habit Formation: Promoting skin care, ergonomic posture, and sustained physical activity for the caregiver. | 135 min |
| Month 3 / Week 11 | Home Session 1 | Nutritional | Habit Consolidation | Vegetable Implementation: Strategies to ensure two servings of vegetables daily. | 15 min |
|  |  | Cognitive | Executive Function | Daily Task Planning: Structuring and sequencing complex daily living activities. | 20 min |
|  |  | Physical | Strength / Mobility | Advanced Trunk Flexo-extension: High-intensity resistance training (3 sets / 10 reps). | Borg: 8. Total: 45 min |
|  | Home Session 2 | All | Functional Nutrition | Culinary Laboratory (Salad Variations): Integrated preparation of nutrient-dense salads with cognitive-motor drills. | Borg: 8. Total: 70 min |
|  | Independent Tasks | All | Procedural Planning | Task Sequencing: Detailed 3-step planning for daily activities; preparation of a recipe from the provided cookbook. | Walk: 30 min (Borg 8), 5x/week |
|  | Group Session | Familiar | Reflection & Closing | Program Synthesis: Final physical activities and reflection on the caregiving journey. | 150 min |
| Month 3 / Week 12 | Home Session 1 | Nutritional | Legume Benefits | Protein Diversification: Benefits of legumes in a neuroprotective diet. | 15 min |
|  |  | Cognitive | Memory III | Working & Episodic Memory: High-complexity recall and information manipulation tasks. | 20 min |
|  |  | Physical | Balance / Coordination | Advanced Flexo-extension: Integrated mobility and stability drills (3 sets / 10 reps). | Borg: 8. Total: 45 min |
|  | Home Session 2 | Nutritional | Recipe Consolidation | Legume Cookbook: Review and final implementation of healthy legume recipes. | 15 min |
|  |  | Cognitive | Memory III | Narrative Recall: Final advanced memory training sessions. | 20 min |
|  |  | Physical | Strength / Aerobic | High-Intensity Static Gait: Final resistance and cardiovascular challenge (3 sets / 8 min). | Borg: 8. Total: 45 min |
|  | Independent Tasks | All | Emotional Memory | Photographic Narrative: Attaching descriptions and emotional context to 3 personal family photos. | Walk: 30 min (Borg 8), 5x/week |
|  | Group Session | Familiar | Graduation | Final Ceremony: Reflections on experiences, graduation, and establishing long-term care sustainability. | 150 min |

Supplementary Table S2. Plan for control group participants by week (infographics)

| **Week** | **Topics** |
| --- | --- |
| 1 | Introduction: Vulnerability of Older Adults to Infections |
| 2 | Community-Acquired Pneumonia (CAP) |
| 3 | Urinary Tract Infections (UTIs) |
| 4 | Influenza and Its Impact on Older Adults |
| 5 | Tuberculosis: Risk Factors in the Elderly |
| 6 | Antimicrobial Resistance in Older Adults |
| 7 | *Clostridioides difficile* and Antibiotic Use |
| 8 | Herpes Zoster: Early Diagnosis and Management |
| 9 | COVID-19: Lessons Learned and Ongoing Prevention |
| 10 | Infection Prevention Through Hygiene and Nutrition |
| 11 | Vaccination in Older Adults: Current Priorities |
| 12 | One-health |

Supplementary Table S3. Participant timeline (SPIRIT figure): schedule of enrolment, interventions and assessments

|  | P | BL | +3m | +6m | +9m |
| --- | --- | --- | --- | --- | --- |
| PATIENT |  |  |  |  |  |
| PRESELLECTION |  |  |  |  |  |
| Geriatric Depression Scale (GDS-15) | X |  |  |  |  |
| Barthel Index of Activities of Daily Living (ADL) | X |  |  |  |  |
| Physical Activity Readiness Questionnaire for Everyone (PAR-Q & YOU) | X |  |  |  |  |
| NEUROPSYCHOLOGICAL ASSESSMENT |  |  |  |  |  |
| Mini-Mental State Examination (MMSE) |  | X | X | X | X |
| Montreal Cognitive Assessment (MoCA) |  | X | X | X | X |
| Hopkins Verbal Learning Test (HVLT) |  | X | X | X | X |
| Symbol Digit Modalities Test (SDMT) |  | X | X | X | X |
| Trail Making Test (TMT-Forms A and B) |  | X | X | X | X |
| Boston Naming Test |  | X | X | X | X |
| Stroop Color and Word Test |  | X | X | X | X |
| Change in the scores of the Rey-Osterrieth Complex Figure Test |  | X | X | X | X |
| Digit Span subtest of the WAIS-III |  | X | X | X | X |
| Token Test |  | X | X | X | X |
| Semantic and Phonemic Verbal Fluency Test |  | X | X | X | X |
| NURSING ASSESSMENT |  |  |  |  |  |
| Measurements of blood pressure - OMROM model HEM-7130 automated monitor |  | X | X | X | X |
| Pulse |  | X | X | X | X |
| Height and waist and hip circumferences - standardized method |  | X | X | X | X |
| Pittsburgh Sleep Quality Index (PSQI) |  | X | X | X | X |
| Bristol Stool Form Scale (BSFS) |  | X | X | X | X |
| Patient Characterization Form (basic information, family, pathological, pharmacological, and surgical history) |  | X | X | X | X |
| Family APGAR |  | X | X | X | X |
| SF-36 Health Survey |  | X | X | X | X |
| Linda Fried Frailty Phenotype |  | X | X | X | X |
| Mini Nutritional Assessment (Long-MNA®) |  | X | X | X | X |
| PHYSIOTHERAPY ASSESSMENT |  |  |  |  |  |
| Dominant handgrip strength - JAMAR 6386 hydraulic analog hand dynamometer |  | X | X | X | X |
| Tinetti Gait and Balance Assessment Tool |  | X | X | X | X |
| Senior Fitness Test |  | X |  |  |  |
| Lawton-Brody Instrumental Activities of Daily Living Scale |  | X | X | X | X |
| FAMILY MEMBER ASSESSMENT |  |  |  |  |  |
| GCPC-UN-D© - sociodemographic characterization of the patient-family study partner |  | X | X | X | X |
| MOS Social Support Survey |  | X | X | X | X |
| Quality of life - SF-36 |  | X | X | X | X |
| Zarit Caregiver Burden Interview |  | X | X | X | X |
| MEDICAL EVALUATION (neurologist and psychiatrist for patients) |  |  |  |  |  |
| Neuroimaging history |  | X |  |  |  |
| Clinical laboratory history |  | X |  |  |  |
| Medical and family history, |  | X |  |  |  |
| Neurological/psychiatric assessment |  | X |  |  |  |

P: preselection, BL: baseline, +3m: follow-up 3 months, +6m: follow-up 6 months, +9m: follow-up 9 months,
